# Sexual function in male cancer survivors is not correlated to sperm quality

**DOI:** 10.1007/s00520-022-06957-z

**Published:** 2022-03-09

**Authors:** Elisabeth Reiser, Anna Lena Zippl, Kilian Vomstein, Elena Strassgschwandter, Susanne Hofer-Tollinger, Germar Michael Pinggera, Bettina Toth

**Affiliations:** 1 Department of Gynecological Endocrinology and Reproductive Medicine, Medical University Innsbruck, Anichstrasse 35, 6020 Innsbruck, Austria; 2 Department of Urology, Medical University Innsbruck, Innsbruck, Austria

**Keywords:** Erectile dysfunction, Gonadotoxic treatment, Infertility, Fertility preservation

## Abstract

**Purpose:**

Both infertility and erectile dysfunction (ED) are known long-term consequences of cancer treatment in young male cancer survivors. In the present study, we aimed to assess whether sperm quality and sexual function in male cancer survivors are associated.

**Methods:**

In this prospective study, *n* = 244 patients male cancer survivors who underwent sperm analysis and cryopreservation between 2008 and 2018 prior to the initiation of gonadotoxic treatment were invited. In total *n* = 50 had a follow-up sperm analysis and completed two questionnaires, the Aging Males’ Symptom Scale (AMS) and the International Index of Erectile Function (IIEF-EF). Differences between the individual parameters were analyzed using the Wilcoxon or Mann Whitney test.

**Results:**

Azoospermia was present in *n* = 16/50 (32.0%) patients at time of follow-up. ED occurred in *n* = 9/43 (20.9%) patients and was observed more frequently in patients with oligo- or azoospermia than in those with normospermia, even though this association was not statistically significant. Sperm parameters (total sperm count, sperm concentration, progressive motility) did not differ between time of cryopreservation and time of follow-up. Mean total, somatic, psychological, and sexual AMS score was 23.6, 9.9, 6.6, and 6.8, respectively. Mean total IIEF-EF score was 27.3, indicating mainly mild ED.

**Conclusions:**

More than one-third of cancer patients suffered from azoospermia, and ED was primarily present in this subgroup. We recommend implementing the screening of sexual dysfunction in the annual sperm testing that should be offered to all men after gonadotoxic treatment. Our study highlights the importance of counseling young cancer patients on both aspects—future infertility and sexual function—prior to treatment and at follow-up visits.

## Introduction

Survival rates of young cancer patients are constantly rising, reaching 5-year survival rates of up to 80–98% [1, 2], depending on cancer type and stage. This creates a new group of patients: cancer survivors who face the long-term consequences of their treatment (surgery, chemotherapy, immunotherapy, and/or radiotherapy), which include transient or permanent infertility [3]. During the last decades, the awareness of clinicians for this problem has risen. As a result, sperm cryopreservation prior to the initiation of gonadotoxic treatment is routinely offered to male cancer patients to fulfill their desire for children in their later lives [4].

However, fertility is only one facet of sexual function that is possibly impacted by cancer therapies, and few studies have aimed to understand the association between cancer and sexual function besides fertility, such as erectile dysfunction (ED). Male cancer survivors frequently suffer from ED [5], which depends on the type of cancer, and treatment regimes, but also age and comorbidities [6–8]. In testicular cancer patients, for example, the rate of ED was reported to be as high as 37% [9]. A Danish testicular cancer cohort study reported different rates and manifestations of ED according to the applied therapies. ED was described in men who underwent radiotherapy, BEP (bleomycin, etoposide, and platinum) chemotherapy with subsequent surgery, or more than one line of treatment. The latter group also reported orgasmic dysfunction, while the group treated with radiotherapy also reported decreased sexual satisfaction [10].

The varying prevalence and manifestation of ED in cancer survivors is partially explained by the multifactorial pathomechanisms involved. Surgery, particularly retroperitoneal lymphadenectomy (RPLND), and radiation may lead to neural damage and impede erection and ejaculation [11]. Due to Leydig cell damage, cancer treatment can further lead to hypoandrogenism, which may impair sexual function, even though the exact correlation needs to be further explored. Low testosterone levels have been reported in up to 26% of testicular cancer survivors [12, 13]. Besides the potential impact on sexual function, male hypoandrogenism also predisposes to the development of metabolic and cardiovascular diseases [14], which are again risk factors for ED. Besides biological mechanisms, the psychological impact of being diagnosed with and treated for cancer is another important—yet often neglected—factor [15].

A possible association between sperm quality and sexual function in male cancer survivors remains unclear. In infertile couples without a history of cancer, ED prevalence has been reported to increase as a function of sperm quality impairment [16]. It is plausible that a similar association exists for cancer survivors. Better understanding the joint occurrence of poor sperm quality and ED in cancer survivors could help clinicians to identify patients at risk.

In the present study, we aimed to assess the association of sperm quality and sexual function in cancer survivors.

## Material and methods

### Study population

This prospective study included patients who had their sperm samples cryopreserved between January 1, 2008, and July 1, 2018, at a tertiary hospital. All patients were invited for a follow-up visit including a novel sperm testing as well as two questionnaires. Initially, sperm cryopreservation had been performed immediately after the diagnosis of malignant or benign diseases that required surgery or, potentially, gonadotoxic treatment. Medical history, sociodemographic parameters, and laboratory data (i.e., age, body mass index (BMI), as well as the levels of thyroid-stimulating hormone (TSH), follicle-stimulating hormone (FSH), and luteinizing hormone (LH)) were obtained. Malignant diseases were classified according to the international guidelines, such as Ann Arbor staging (for hematological malignancies) or Union Internationale Contre le Cancer stadium (UICC), for testicular malignancies. The study was approved by the Local Review Board (IRB Number 1016/2020), and all patients gave written informed consent before recruitment.

### Questionnaires

Two questionnaires on health-related quality of life (HRQoL) and sexual functioning—the Aging Males’ Symptom Scale (AMS) [17, 18] and the International Index of Erectile Function (IIEF-EF) [19, 20]—were completed by the participants at the time of follow-up sperm sampling. The AMS (a) assess symptoms of aging (independent from those which are disease-related) between groups of males under different conditions, (b) evaluates the severity of symptoms over time, and (c) measures changes pre- and post-androgen replacement therapy. It was developed in response to the lack of standardized scales to measure the severity of aging symptoms and their impact on HRQoL in males, specifically. The AMS scale consists of 17 items in three domains (psychological, somatic, and sexual) on a scale of 1–5. The total score for each of the domains is based on adding up the scores of the items of the respective domain. The cumulative score ranges from 17 to 85 points. The severity of the symptoms is defined as: no/low (17–26 points), mild (27–36 points), moderate (37–49 points), and severe (≥ 50 points). Sexual function can be evaluated by the IIEF-EF. The six items on the IIEF-EF include detailed questions concerning erection frequency, erection firmness, penetration ability, maintenance frequency, maintenance ability, and erection confidence. Participants needed to report sexual activity at least once during the 4 weeks before responding to the questions. Each item was based on a 5-point Likert scale. For each subject, the responses of all six items of the IIEF-EF were summed to a total EF score, with a range from 6 to 30. Scores lower than 26 indicated the presence of erectile dysfunction. The severity of ED was classified into five categories: no ED (EF score 26 to 30), mild (EF score 22 to 25), mild to moderate (EF score 17 to 21), moderate (EF score 11 to 16), and severe (EF score 6 to 10). Moreover, patients were asked concerning family planning including questions about fatherhood before and after gonadotoxic treatment and the total number of children.

### Sperm analysis

Initial sperm samples were obtained by masturbation-induced ejaculation before gonadotoxic treatment. The time of ejaculation abstinence was recorded with the recommendation of an interval of at least 2–3 days. However, for urgent cases, sperm storage was performed regardless of the abstinence. Men with low total sperm counts were advised to provide one or more additional samples, again with an optimal abstinence time of 2–3 days in order to obtain sufficient numbers of sperms for cryopreservation. At the time of follow-up, another sperm sample was obtained by masturbation. Normozoospermia was defined for all samples in accordance with the 2010 WHO criteria (sperm concentration ≥ 15 million/mL, progressive motility ≥ 32%, and ≥ 4% normal morphology). Sperm samples obtained before 2010 (2008–2010) were evaluated following the 1999 WHO criteria and adjusted to the criteria of 2010.

### Statistics

Analysis of variance (ANOVA) was performed for normally distributed raw data, which was presented as mean ± standard deviation (SD). For non-normal data distribution, the differences between the individual parameters of the groups were analyzed using the Wilcoxon or Mann Whitney test and presented as median (interquartile range (IQR)). To prevent alpha error accumulation, a Bonferroni correction was applied for multiple comparisons. To compare means, the paired t-test was applied. The Spearman’s rank correlation analysis was used to identify correlations between different parameters. A significance level of *α* = 0.05 was assumed for all statistical evaluations. The statistical analysis was conducted using IBM SPSS Statistics for Windows, Version 26.0 (IBM Corp., released in 2019; Armonk, NY: IBM Corp).

#### Results

In total, *n* = 244 male cancer patients were eligible for the study inclusion as they underwent sperm cryopreservation before gonadotoxic treatment. At the time of screening, *n* = 27 patients had died of their underlying disease, and *n* = 77 were living abroad or in another state, leaving *n* = 126 patients for possible participation. Finally, *n* = 50 (39.6%) male cancer survivors followed the invitation to participate in this study and a follow-up visit including sperm sampling and two questionnaires. Demographic data are shown in Table 1. Median age (IQR) at time of cancer diagnosis and follow-up was 25.0 (19.8–29.0) and 30.0 (26.8–35.0) years, respectively, resulting in a median follow-up time of 64.0 (45.3–106.8) months. In sum, *n* = 24 (48.0%) patients suffered from hematological, nineteen (38.0%) from testicular, and seven from other malignancies (e.g., solid tumors, sarcoma)*.* Most of the patients (84.0%) received chemotherapy, and 16.0% underwent radiotherapy or surgery. Out of the 19 patients with testicular malignancy, 15 underwent hemicastration alone, while four patients additionally underwent retroperitoneal lymphadenectomy. Two patients with retroperitoneal lymphadenectomy showed azoospermia without signs of erectile dysfunction at time of follow-up. Of note, these patients comprise a very small group not allowing interpretation of results. Patients with other malignancies underwent orthopedic surgeries of the lower extremity (*n* = 2) or no surgery (*n* = 5), whereas patients with hematological diseases underwent no surgery.

**Table 1:** Demographic data

Parameter	
Age at diagnosis (years)^a^	25.0 (19.8–29.0)
Age at follow-up (years)^a^	30.0 (26.8–35.0)
Follow-up time (months)^a^	60 (45.3–106.8)
BMI (kg/m^2^)^a^	22.7 (20.2–25.5)
Smoking	
Yes	11 (22.4)
No	38 (77.6)
Alcohol consumption	
Yes	16 (67.3)
No	33 (32.7)
Type of malignancy^b^	
Hematological malignancy	24 (48.0)
Testicular malignancy	19 (38.0)
Others	7 (14.0)
Chemotherapy^b^	
Yes	42 (84.0)
No	8 (16.0)
Radiation^b^	
Yes	10 (20.0)
No	40 (80.0)
Fatherhood^b^	
Before diagnosis	2 (4.8)
After diagnosis	8 (19.0)
Desire to have a child at of follow-up	25 (61.0)
Spontaneous pregnancy, *N*	6
ART, *N*	3
AMS (44)	
Total^c^	23.6 (9.3)
Subscales ^c^	
Somatic (7–35)	9.9 (4.5)
Psychological (5–25)	6.6 (2.5)
Sexual (5–25)	6.8 (3.5)
AMS categories, N (%)	
No/low symptoms	37 (84.1)
Mild symptoms	3 (6.8)
Moderate symptoms	3 (6.8)
Severe symptoms	1 (2.3)
IIEF-EF total^c^ (43)	27.3 (3.2)
Erectile dysfunction^b^	
Yes	9 (20.9)
No	34 (79.1)
Azoospermia at time of follow-up^b^	16 (32.0)

^a^Median (IQR)

^b^*n* (%)

^c^Mean (SD)

The mean total AMS score was 23.6 (9.3) indicating no/mild symptoms of aging. 15.9% of men experienced age-related symptoms. Mean somatic, psychological, and sexual AMS score was 9.9 (4.5), 6.6 (2.5), and 6.8 (3.5), respectively. Mean total IIEF-EF score was 27.3 (3.2), indicating the presence of ED in *n* = 9/43 (20.9%) of patients. We did not find a significant difference in the presence of ED between patients with hematological malignancies (*n* = 4/20, 20.0%) and patients with testicular malignancies (*n* = 4/17, 23.5%, *p* = 0.55). Also, there was no difference between normospermic and azoospermic patients with regard to ED/age-related symptoms.

Azoospermia and pathospermia were present in *n* = 16 (32.0%) and *n* = 21 (42.0%), respectively, according to the WHO 2010 criteria at the time of follow-up. Significantly more patients showed pathospermia at time of follow-up (*n* = 21, 42.0%) versus *n* = 13 (28.3%) at time of diagnosis (*p* = 0.006). When dividing the patients into the two subgroups normo- and pathospermia, we could not identify any significant differences in the presence/grade of ED, scores in the AMS, or other clinical parameters such as BMI, fatherhood at the time of diagnosis, or follow-up (Table 2). When comparing the single sperm parameters between the time of diagnosis and time of follow-up, no significant differences were identified (Table 3).

**Table 2:** Sexual functioning and quality of life in cancer survivors

Parameter	Normospermia (*N* = 29)	Pathospermia (*N* = 21)	*p*-value
AMS subscales^a^			
Somatic (7–35)	9.7 (3.5)	10.3 (6.3)	0.89^1^
Psychological (5–25)	6.7 (1.9)	6.5 (3.7)	0.37^1^
Sexual (5–25)	6.7 (2.9)	6.9 (4.6)	0.79^1^
AMS categories ^b^			0.64^2^
1	26 (86.6)	14 (82.6)	
2	2 (6.7)	1 (5.8)	
3	2 (6.7)	1 (5.8)	
4	0	1 (5.8)	
IIEF-EF total^a^	27.7 (4.0)	26.3 (4.7)	0.40^1^
IIEF-EF categories^b^			0.35^2^
No erectile dysfunction	23 (85.2)	11 (68.8)	
Erectile dysfunction	4 (14.8)	5 (31.2)	
Mild erectile dysfunction	2	4	
Mild to moderate erectile dysfunction	1	1	
Moderate erectile dysfunction	0	0	
Severe erectile dysfunction	1	0	

^1^Mann Whitney U test

^2^Chi-quadrat

^a^Mean (SD)

^b^*n* (%)

^c^Mean (SD)

**Table 3:** Sperm quality before and after gonadotoxic treatment in cancer patients after a median follow-up time of 64.0 months

	Time of diagnosis	Time of follow-up	*p*-value
Concentration (Mio/ml), mean (SD)	26.6 (20.6)	23.3 (25.7)	0.43^a^
Total count (Mio), mean (SD)	67.2 (50.9)	74.3 (91.8)	0.64^a^
Progressive motility, mean (SD)	43.7 (17.6)	50.5 (15.5)	0.06^a^
Pathospermia, *N* (%)	13 (28.3)	21 (42.0)	0.006^b^

^a^Wilcoxon test

^b^Chi quadrat test

While initially only *n* = 2/42 (4.8%) patients reported fatherhood, *n* = 8/42 (19.0%) patients had a child at time of follow-up. At the same time, *n* = 25/41 (61.0%) patients expressed a desire to have children. Six patients achieved a spontaneous pregnancy within 5 years after cryopreservation, and three patients used the cryopreserved sperm for ART at our department.

## Discussion

Our study shows a high prevalence of ED in young male cancer survivors, affecting more than 20% of the patients in our cohort. To our knowledge, our study is the first to assess the correlation between sperm quality and sexual function in cancer survivors. ED was observed more frequently in patients with oligo- or azoospermia than in those with normospermia, even though this association was not statistically significant. Almost one-third of the patients suffered from azoospermia at the time of follow up. At the same time, more than 60% expressed the desire to have children, emphasizing the importance of routine sperm cryopreservation prior to the initiation of gonadotoxic treatment.

In previous studies, the frequency of sexual dysfunction in cancer survivors varies widely. In our cohort, hematological malignancies accounted for almost half of the cases, showing an ED rate of 20%, similar to the rate described by Eeltink et al. for male Hodgkin lymphoma survivors [7]. In testicular cancer survivors, the prevalence of ED was reported to be higher, reaching up to 37% [9]. In our study, in this subgroup, ED occurred in 23.5%, confirming a higher prevalence in testicular cancer survivors, even though the difference between the two diagnostic groups did not reach statistical significance. The lower prevalence in our testicular cancer patients might be related to a different composition regarding cancer stage.

Another important factor influencing the probability of developing ED is the therapeutical approach chosen. For example, different chemotherapeutical regimens seem to harm sexual function to varying extent. Eeltink et al. described lower IIEF-EF scores in male Hodgkin Lymphoma survivors treated according to the BEACOPP regimen than in those treated with doxorubicin, bleomycin, vinblastine, and dacarbazine [7]. In our cohort, 84% of all patients received chemotherapy, most frequently including cyclophosphamide. Comparing patients who had received a regime containing cyclophosphamide or cisplatin with those who were treated according to regimes free from these agents, we could not observe a difference in the ED rate, being 25% in both groups. Of note, the sample size might be too small for further interpretation.

Independently of cancer type, most studies described a correlation between age at cancer diagnosis and the prevalence or degree of sexual dysfunction, with an increased risk of persisting sexual problems for older patients [6, 8, 21]. This may partially be explained by the fact that sexual activity in general decreases with advancing age. Moreover, older patients suffer from a greater number of comorbidities, which may also influence sexual function directly [8, 21]. However, since our cohort consisted primarily of young patients under the age of 35 years, cases of ED observed in this study are unlikely to be driven by patient’s age or pre-existing comorbidities.

Besides the rather few studies demonstrating sexual dysfunction in cancer survivors, it has long been known that cancer treatments are potentially gonadotoxic and may lead to infertility. Nevertheless, the possible correlations between these two aspects of sexuality—e.g., sexual function and sperm quality—in cancer survivors have not been studied in detail so far. This is surprising, especially as in infertile couples without cancer history, an association between severity of sperm quality impairment and sexual function has been described by Lotti et al. in 2016 [16]. In fact, our data showed a similar trend for male cancer survivors, as those with oligo- or azoospermia showed lower ED scores than those with normospermia.

In our study, we primarily aimed to assess whether there is an association between sperm quality and sexual function in cancer survivors. The observed relationship is multifaceted, with several potential causes contributing. These include damage of testosterone-producing Leydig cells or local nerves, concomitant with germ cell damage, both induced by chemo- and radiotherapy, but also cancer localization and stage as well as psychological aspects.

In the study by Lotti et al., participants knew about their semen quality before answering the questionnaires assessing sexual function. This is a potential bias, as sexual function is strongly associated with psychological aspects. For infertile patients without a cancer history, sexual dysfunction following diagnosis of male factor infertility has been reported [22, 23]. The strong psychological association between fertility and sexuality is also highlighted by an interview study by Frederick et al., in which more than half of the included male childhood cancer survivors spontaneously expressed concerns about their fertility when asked about their sexual function [15]. Other studies found a higher incidence of male sexual dysfunction in patients suffering of anxiety disorders, in particular post-traumatic stress disorder (PTSD) [24–27]. Cancer has lately been recognized as one possible stressor inducing PTSD, with a prevalence of PTSD between 7 and 75% among cancer patients [28–31]. On the other hand, there is evidence to support that stress has a negative effect on semen quality in healthy men [32, 33]. In our study, we tried to reduce the bias linked to the psychological aspects by informing the participants about the results of their follow-up sperm analysis only once they had completed the two questionnaires. Nevertheless, they had been informed about their semen quality at time of cryopreservation, a knowledge that may have influenced their ED scores. It remains therefore unclear whether poor semen quality and ED are independently associated or whether the psychological consequences of knowing about one’s infertility led to ED. Future studies should therefore concentrate on elucidating the exact pathophysiological inter-relations between stress, semen quality, and erectile dysfunction, including more specific questionnaires on patients’ psychological status.

A strength of the study is that all sperm analyses were performed in the same IVF laboratory according to the WHO criteria, reducing the inter-observer variability. With AMS and IIEF-EF, we used validated questionnaires to evaluate ED. Reported scores have a high probability to reflect the long-term outcomes of these young patients, as the median follow-up time was as long as 5 years after cancer diagnosis and sperm cryopreservation.

A limitation of our study consists in the small sample size, which did not allow us to reach statistical significance for small effect sizes. Another aspect that—like in most other studies concerning ED in cancer survivors—could not be assessed regards sexual function before cancer diagnosis and treatment. Therefore, it remains unclear to what extent the reported sexual dysfunction is imputable to the disease and/or therapy. While in previous studies testicular cancer patients have been reported to show lower sperm count already at pre-treatment sperm analysis [34], little is known about the sexual function at that point. In order to reach a better understanding of the causal relationship between cancer, cancer treatment, and ED, future studies should assess the prevalence of ED at cancer diagnosis and compare it with the ED rates after completion of treatment.

Another possible limitation of our study is given by the lack of an age-matched control group. In fact, the reported prevalence of ED in the general population varies widely as a function of sociocultural context. In Germany, presenting a comparable sociocultural context, the prevalence of erectile problems in the general population aged between 26 and 35 years was reported to be as low as 7% [35]. The large difference to the prevalence we observed in cancer survivors of the same age group highlights the detrimental impact of cancer diagnosis and treatment on sexual function.

## Conclusion

Overall, our study highlights the importance of counseling young cancer patients not only with regard to potential infertility, but also taking potential sexual problems into account. Given the high prevalence and the importance for the quality of life, clinicians should screen for sexual dysfunction in all male cancer survivors, especially in those presenting with oligo- or azoospermia. We recommend implementing the screening of sexual dysfunction in the annual sperm testing that should be offered to all men after gonadotoxic treatment. Future studies should investigate the association of semen quality and sexual function in larger cohorts, allowing to control for age, type of cancer, stage at diagnosis, and applied treatment.

## Data Availability

The datasets generated during and/or analyzed during the current study are available from the corresponding author on reasonable request.

## Code availability

The code generated during and/or analyzed during the current study are available from the corresponding author on reasonable request.
